# Myeloid Cells in the Tumor Microenvironment: Modulation of Tumor Angiogenesis and Tumor Inflammation

**DOI:** 10.1155/2010/201026

**Published:** 2010-05-16

**Authors:** Michael C. Schmid, Judith A. Varner

**Affiliations:** Moores UCSD Cancer Center, University of California, San Diego, 3855 Health Sciences Drive, La Jolla, CA 92093-0912, USA

## Abstract

Myeloid cells are a heterogeneous population of bone marrow-derived cells that play a critical role during growth and metastasis of malignant tumors. Tumors exhibit significant myeloid cell infiltrates, which are actively recruited to the tumor microenvironment. Myeloid cells promote tumor growth by stimulating tumor angiogenesis, suppressing tumor immunity, and promoting metastasis to distinct sites. In this review, we discuss the role of myeloid cells in promoting tumor angiogenesis. Furthermore, we describe a subset of myeloid cells with immunosuppressive activity (known as myeloid-derived suppressor cells). Finally, we will comment on the mechanisms regulating myeloid cell recruitment to the tumor microenvironment and on the potential of myeloid cells as new targets for cancer therapy.

## 1. Introduction

Angiogenesis, the growth of new blood vessels, occurs at different stages during embryonic development, physiological processes such as wound healing and reproduction, and numerous diseases, including inflammation, tumor progression, and metastasis [1]. The human immune system is composed of an innate and an adaptive branch. They both play a key role in maintaining homeostasis within our organism. The innate immune system is mainly composed of myeloid lineage cells, such as macrophages, neutrophils, and mast cells [2]. Under nontumor conditions, these cells provide the first line of protection against pathogens. Importantly, during tumor progression, myeloid cells are implicated in promoting tumor angiogenesis, causing resistance against antiangiogenic therapies in cancer, and suppressing the immune response during cancer [3–5].

## 2. Angiogenesis and Vasculogenesis during Tumor Growth

### 2.1. Angiogenesis

Neovascularization, the formation of new blood vessels, plays important roles in development, inflammation, and wound repair. Mammalian cells require oxygen and nutrients for their survival and are therefore located within 100 to 200 *μ*m of blood vessels, the diffusion limit of oxygen. In 1971, Dr. Judah Folkman observed that neovascularization occurs around tumors and proposed that new blood vessel growth is necessary to supply nutrients and oxygen to tumor cells during exponential tumor growth [6]. These observations stimulated an intensive search for the mechanisms regulating tumor angiogenesis. It is now known that new blood vessels originate from preexisting vessels by activation, proliferation and migration of endothelial cells through a process named “angiogenesis” [4]. Specific growth factors, such as vascular endothelial growth factor (VEGF) and basic fibroblast growth factor (bFGF), stimulate the proliferation and migration of naturally quiescent endothelial cells, resulting in the formation of new vessel structures during embryonic development and tumor growth [7]. Although tumor cells were first thought to drive the cellular events underpinning tumor angiogenesis and growth, considerable evidence has now emerged for the central role of tumor infiltrating myeloid cells such as monocytes, macrophages, and neutrophils in this phenomenon [8–12]. 

### 2.2. Vasculogenesis

Vasculogenesis is the coalescence of new blood vessels from individual endothelial cells or progenitor cells. Until recently, vasculogenesis was thought to be restricted to the formation of the initial vascular tree during embryonic vascular development. In 1997, Asahara et al., [13] isolated mononuclear cells from human peripheral blood that were enriched for expression of the hematopoietic stem cell marker CD34 [13]. Upon culture in endothelial growth media, these cells expressed endothelial lineage markers, such as CD31, Tie2, and VEGF receptor 2 (VEGFR2), and incorporated into blood vessels in ischemic tissues. These cells were therefore described as bone marrow derived endothelial progenitor cells (EPCs). Subsequent studies described a VEGFR2 and AC133 expressing subpopulation of these CD34 positive circulating cells that could form endothelial colonies in vitro [14, 15]. This suggested that EPCs are able to differentiate into endothelial cells and that such cells are incorporated into sites of active angiogenesis including ischemia, tumor angiogenesis, and metastasis in adult organisms [16, 17]. Since then, the study of circulating EPCs has generated considerable interest and controversy. Different markers, methods, and different kinds of cancer models used to identify EPC probably contributed to the widely divergent reports of the level of incorporation of these cells into newly formed tumor blood vessels [5]. These levels ranged from highs of 20% to 50% to lows of 5% or even less, the lower levels being more common [18–20].

## 3. M1 and M2 Tumor Associated Macrophages

### 3.1. Classical and Alternative Activation

Monocytes and macrophages belong to the myeloid cell lineage and derive from myeloid progenitor cells. These precursor cells are located in the bone marrow; upon maturation, monocytes are released into the bloodstream. Circulating blood monocytes migrate into tissues where they differentiate into resident tissue macrophages. 

**Figure 1:**
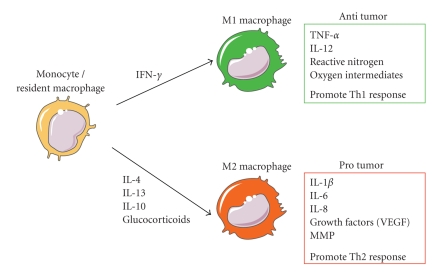
Cytokines produced in the tumor microenvironment can give rise to macrophages with distinct physiologies. Classical activated macrophages (M1) arise in response to interferon *γ* (IFN-*γ*). M1 macrophages elicit tissue disruptive reactions by producing tumor necrosis factor *α* (TNF-*α*), interleukin 12 (IL-12), reactive nitrogen, and oxygen intermediates. M1-activated macrophages are part of the polarized Th1 response. M2 macrophages are generated in response to various stimuli, including IL-4, IL-13, IL-10, and glucocorticoids. Tumor-associated macrophages have properties of M2-activated cells. They express many proangiogenic and angiogenic modulatory factors such as IL-1*β*, IL-6, IL-8, vascular endothelial growth factors (VEGFs), and matrix metalloproteinases (MMPs). M2 macrophages are part of the Th2 response.

Macrophages are activated in response to environmental signals, including microbial products and cytokines. Activated macrophages can be divided into M1 (classical activated) and M2 (alternative activated) phenotype (Figure 1) [21]. Classical activation occurs in response to bacterial moieties such as lipopolysacharide (LPS) and immune stimuli such as interferon-*γ* (IFN-*γ*). M1 macrophages mediate resistance against intracellular parasites and tumors and elicit tissue disruptive reactions by secreting tumoricidal agents such as tumor necrosis factor *α* (TNF-*α*), interleukin-12 (IL-12), reactive nitrogen (iNOS), and oxygen intermediates (ROS). In addition, M1 macrophages promote T-helper-1 (Th1) responses. By contrast, M2 activated macrophages come in different varieties depending on the eliciting signals, which include IL-4, IL-13, IL-10, and glucocorticoid hormones. In general, M2 macrophages have an immune suppressive phenotype and release cytokines including IL-10 that promote a Th2 immune response [22–24]. Macrophages in tumors—usually termed tumor-associated macrophages (TAMs)—often express the M2 phenotype. However, recent evidence suggested that the phenotype of TAM varies with the stage of tumor progression. M1 macrophages are often abundant in chronic inflammatory sites, where tumors are initiated and start to develop. Then the macrophages switch to an M2-like phenotype as the tumor begins to invade, vascularize, and develop [25].

### 3.2. Proangiogenic Phenotype

M2-like TAMs release a number of potent proangiogenic cytokines, such as VEGF-A, VEGF-C, TNF-*α*, IL-8, and bFGF [26, 27]. Additionally, these TAMs also express a broad array of proteases known to play roles in the angiogenic process. These proteases include urokinase-type plasminogen activator (uPA), the matrix metalloproteinases MMP-2, MMP-7, MMP-9, and MMP-12, and elastase [28, 29]. uPA and MMP support angiogenesis by remodeling and breaking down the extracellular matrix (ECM). Degradation of ECM leads to the mobilization of growth factors and facilitates the migration of vascular cells into new environments [30–32]. Strong correlations are observed between TAM densities and vascular densities in many human tumor types, suggesting that TAMs regulate neovascularization. Importantly, high TAM densities are indicative of poor prognoses in breast, prostate, ovarian, and cervical cancers [33–35].

## 4. Myeloid Derived Suppressor Cells

### 4.1. Heterogeneous Family

Besides promoting angiogenesis, a subset of myeloid cells can facilitate tumor growth by their ability to downregulate the immune response against cancer cells. These so-called myeloid derived suppressor cells (MDSCs) are a heterogeneous population of cells that consist of myeloid progenitor cells and immature myeloid cells (IMCs). In healthy individuals, IMCs are generated in the bone marrow. They quickly differentiate into mature granulocytes, macrophages, or dendritic cells (DCs). In contrast, in pathological conditions such as cancer, a partial block in the differentiation of IMCs into mature myeloid cells occurs, which results in the expansion of the MDSC population. MDSCs can be found in the bone marrow (BM), spleen, and tumor sites and have been identified in most patients and in experimental mice with tumors based on their ability to suppress T cell activation [36].

MDSCs lack the expression of cell surface markers that are specifically expressed on monocytes, macrophages, or DC. In mice MDSCs are uniformly characterized by the expression of the cell surface molecules detected by antibodies to Gr1 and CD11b. Gr1 includes the macrophage and neutrophil markers Ly6C and Ly6G, respectively, whereas CD11b (also known as integrin *α*M) is characteristic for the myeloid-cell lineage. In recent years, several other surface molecules have been used to identify additional subset of suppressive MDSC, including CD80 [37], CD115 (also known as macrophage colony-stimulating factor (M-CSF) receptor, and CD124 (IL-4 receptor alpha chain (IL-4Ra)) [38].

In addition, nuclear morphology has also been used to characterize mouse MDSC. MDSCs that are mononuclear are considered “monocytic” and are typically CD11b^+^ Ly6G^+/−^ Ly6C^high^, whereas those with multilobed nuclei are “granulocytic/neutrophil-like” and are CD11b^+^Ly6G^+^Ly6C^−^ [39, 40].

In cancer patients MDSCs are typically defined as CD11b^+^CD14^neg^, cells that express the common myeloid marker CD33 but lack the expression of markers of mature myeloid and lymphoid cells, and of the MHC class II molecule HLA-DR [41, 42]. In addition MDSCs have also been identified within a CD15^+^ population in human peripheral blood [43]. 

### 4.2. Mechanism of Immune Suppression by MDSC

Several mechanisms have been associated with the immunosuppressive effects of myeloid cells, including secretion of immunosuppressive cytokines, upregulation of nitric oxide (NO), generation of ROS, and increased activity of L-arginase [44].

**Arginase, iNOS, ROS, and COX2:**

L-arginine plays a critical role in the immunosuppressive activity of MDSC. T-cell proliferation and activation depends on the availability of L-arginine. L-arginine is a nonessential amino acid and is a substrate for two enzymes, inducible NO synthase (iNOS or NOS2) and arginase 1. MDSCs express both enzymes at high levels [36]. Recent data suggests that the increased activity of arginase 1 and iNOS in MDSC leads to enhanced L-arginine catabolism, which results in a reduction or depletion of L-arginine in the microenvironment. The lack of L-arginine results in inhibition of T-cell function [41, 45].

MDSC-produced ROS inhibits CD8^+^ T cell by catalyzing the nitration of the TCR and thereby preventing T cell peptide-MHC interactions [46]. In addition, several known tumor-derived factors, such as TGF-*β*, IL-3, IL-6, IL-10, platelet-derived growth factor (PDGF), and granulocyte macrophage colony stimulating factor (GM-CSF), can induce the production of ROS by MDSC [36, 47].

Cyclooxygenase-2 (COX2) is a key factor in the activation of MDSC, because it regulates the expression of arginase 1, iNOS and prostaglandin E2 (PGE2). PGE2 and COX2 are produced by many tumors and are major contributors to the inflammatory milieu [48]. PGE2 was also shown to upregulate CD11b^+^CD14^−^CD15^+^ MDSC in patients with renal cancer [41]. Therefore, elevated PGE2 levels were associated with higher levels and more suppressive MDSC. COX2 inhibitors have proven clinical applications for the treatment of colon cancer and intestinal polyposis [49].

**Cytokines:**

MDSC-derived cytokines can suppress antitumor immunity. Secretion of the type 2 cytokine IL-10 downregulates the production of the type 1 cytokine IL-12 in macrophages. In addition, IL-10 and VEGF inhibit the maturation of DC [50]. TGF-*β* has also been associated with MDSC immune suppressive functions. In fibrosarcoma and colon carcinoma tumor models, MDSC produced TGF-*β* in response to IL-13 stimulation, which resulted in decreased tumor immunosurveillance of cytotoxic T-cells [51, 52].

## 5. Various Protumorigenic Myeloid Subpopulations

In recent times, most studies have analyzed the role that TAM and MDSC have on tumor angiogenesis and progression. However, there is now increasing evidence to show that various other myeloid subpopulations, such as Tie2 expressing monocytes, neutrophils, eosinophils, mast cells, and dendritic cells, also actively participate in these processes. In this paragraph we briefly discuss the likely mechanisms by which these cells driving tumor angiogenesis and progression.

### 5.1. Tie2 Expressing Monocytes

De Palma et al., [53] recently identified a distinct lineage of myeloid cells that can be distinguished from other monocytes by their expression of the angiopoietin receptor Tie2 [53]. Although Tie2 is broadly expressed on vascular endothelial cells and generally regarded as an EC specific marker, Tie2 expressing monocytes (TEMs) are distinct from ECs and do not incorporate in the tumor endothelium. TEMs are a small monocyte subset that circulate in the mouse and human peripheral blood and appear to be preferentially recruited to tumors and other sites of angiogenesis [53]. In mouse blood, TEMs express CD45, the pan leukocyte marker, and CD11b, but do not express Gr1 (Ly6G/C), which is detected on granulocytes, DC, and MDSC. TEMs are a subset of tumor infiltrating F4/80^+^ macrophages. In distinct tumor areas, TEM may account up to 30% of the total F4/80 macrophages [54, 55]. The close proximity of some TEMs to the tumor vasculature suggested to De Palma and colleagues that these cells might contribute to the regulation of tumor angiogenesis. The specific elimination of TEM by suicide gene strategy in mouse tumor models inhibited tumor angiogenesis. Interestingly, ablation of TEM did not affect the recruitment of TAM or neutrophils into these tumors, suggesting that, rather than being precursors of TAM, TEMs comprise a distinct monocyte subpopulation with potent proangiogenic activity. However, it is not clear whether TEM and TAM derive from a common monocytic precursor, or whether tumor microenvironmental factors can induce TAM to acquire a “TEM phenotype” or vice versa. It was suggested that TEMs stimulate angiogenesis by expressing the potent proangiogenic molecule bFGF (although the actual release of this growth factors has yet to be demonstrated) [53].

### 5.2. Neutrophils

Neutrophils are phagocytic, polymorphonuclear cells and are the most abundant subpopulation of leukocytes in the blood and are principally involved in acute inflammatory response to invading microorganisms. Increases levels of neutrophils have been observed in patients with gastric, colon, and lung cancer [56, 57]. In humans, neutrohils can be identified by the cell surface marker CD66b (also known as CEACAM8), or by the cytoplasmic marker myeloperoxidase (MPO) coupled with cell morphology. In murine tumors, Gr1^+^ cells are usually considered to be neutrophils or cells derived from neutrophil precursors. However, it should be noted that murine MDSCs also express Gr1^+^ [36].

The mechanism by which tumor-associated neutrophils mediate or modulate tumor angiogenesis has not been fully elucidated. Tumor-associated neutrophils are a major source of MMP9 (along with macrophages and mast cells) in various murine tumor models and so could promote angiogensis by releasing potent angiogenic factors such as VEGF that are usually sequestered in the ECM [58]. In addition, TNF*α*-stimulated neutrophils undergo degranulation and thereby releasing their intracellular VEGF storage, which subsequently induces endothelial cell proliferation and tube formation in vitro [59]. Recently Fridlender et al. [11] described that tumor-associated neutrophils (TANs) can be polarized in the tumor microenvironment into N1 and N2 phenotype similar as described previously for tumor-associated macrophages. Thereby, within the tumor microenvironment, TGF-*β* induced and maintained a population of TAN with an N2 tumor-promoting phenotype. 

### 5.3. Eosinophils

Eosinophils are characterized by the expression of CCR3 and CD125. They are multifunctional leukocytes implicated in the pathogenesis of numerous inflammatory processes including parasitic helminths infections and allergic diseases [60]. Increased numbers of eosinophils have been reported for several human tumors including oral squamous cell carcinoma, gastrointestinal tumors, Hodgkin lymphoma, and nasopharyngeal carcinoma [61–64]. The highly potent and selective eosinophil chemoattractant CCL11 (eotaxin), which binds to CCR3, was described to mediate the recruitment of eosinophils to the tumor microenvironment [65]. The role of eosinophils in the tumor microenvironment remains unclear. Accumulation of eosinophils in the necrotic region suggests that eosinophils may promote necrosis and might have antitumor activity [66]. Alternatively, there is evidence to suggest that eosinophils recruited to tumor sites can influence angiogenesis. Eosinophils contain VEGF in their secretory granules, which are rapidly secreted upon activation with IL-15 [67]. In addition, TNF*α*-stimulated eosinophils release proangiogenic factors like bFGF, IL-6, IL-8, PDGF, and MMP9 [68]. However, the release of proangiogenic factors of IL-15 and TNF*α*-stimulated eosinophils has only been observed in vitro and has yet to be confirmed in tumors.

### 5.4. Mast Cells

Mature mast cells (MCs) populate most tissues but are found in highest numbers in the skin, airways, and digestive tract, where they are thought to act as a first line of defense against infiltrating pathogens and parasites. MCs also have an important role in generating and maintaining innate and adaptive immune responses as well as the development of autoimmune disorders and tolerance. MCs are usually identified by basic Giemsa or toluidine blue staining, expression of cell surface markers such as C-kit receptor (CD117) in human and CD34 in mice, or stored cytoplasmic molecules including tryptase and chymase [69]. MC originate from the bone marrow as immature cells and migrate to peripheral tissues where they mature in situ. Mast cells are now recognized as an early and persistent infiltrating cell type in many tumors, often entering before significant tumor growth and angiogenesis occurred. Mast cells accumulate at the boundary between healthy tissues and malignancies and are often found in close association with blood vessels within the tumor microenvironment. They express many proangiogenic compounds such as VEGF, bFGF, MMP9, TGF-*β*, TNF*α*, and IL-8. In several human tumors increased MC density positively correlates with increased microvessel density and in some cases, with poor prognosis [70].

### 5.5. Dendritic Cells

Dendritic cells (DCs) are specialized antigen presenting cells that acquire, process, and present tumor-associated antigens to T-cells for the induction of antigen-specific tumor immune response. Two distinct populations of DC exit in mouse and human tissues: (i) myeloid DC (MDC) and (ii) plasmacytoid DC (PDC). MDCs express CD11c and CD33 and lack CD45R, CD123, and Lin, whereas PDCs are CD123^+^, CD45R^+^, CD4^+^, CD11c^−^, ILT3^+^, ILT1^−^, and Lin^−^ [71]. MDCs originate in the bone marrow as immature cells (iDC) that lack the classical mature DC markers, CD1a, CD83, CD40, and CD86. Once they process foreign antigen, they become activated, undergo maturation, and migrate to lymphoid tissue where they initiate activation of antigen-specific T cells [72].

By their potential capacity to activate tumor-specific T-cell responses, DC play an important role in cancer immunosurveillance. Interestingly, circulating and tumor-infiltrating DCs from cancer patients appear to be phenotypically and functionally defective. Several tumor-derived factors have been shown to be responsible for systemic and local DC defects [73]. Beside the vast majority of reports of MDC in cancer focusing on their suppressed immunoregulatory function, it has become apparent that iDCs also promote tumor neovascularization. For example, Conejo-Garcia and colleagues [74] described a mechanism of tumor vasculogensis mediated by DC precursors. *β*-defensin mediated recruited of DC precursors to tumors enhanced tumor vascularization and growth in the presence of increased VEGF-A expression. Thereby VEGF-A induced the simultaneous expression of both, endothelial and DC markers, on DC precursors and the DC precursors underwent endothelial-like specialization. These cells were termed vascular leukocytes (VLCs) and are highly present in human ovarian carcinomas. Depending on the milieu, VLCs can assemble into functional blood vessels or act as antigen-presenting cells [75].

A recent report underlined the important role of immature DC during tumor vascularization [76]. In this study only tumor cells implanted with immature DC, but not with mature DC, revealed increased neovascularization and growth. In addition, complete depletion of DC in a transgenic CD11c^+^DTR-Tg mice model abrogated angiogenesis in bFGF loaded Matrigels and inhibited the growth of intraperitoneally injected B16 melanoma cells (although the tumor model used in this study is uncommon). 

Beside the role of immature DC/VLC in vasculogenesis, immature DC might also promote angiogenesis. A recent report showed that human iDCs upregulate proangiogenic cytokines such as VEGF and IL-8 on exposure to severe hypoxia in vitro [77]. Beside their proangiogenic role, VEGF and IL-8 are also immunosuppressive cytokines capable to inhibit DC maturation and so might act in an autocrine as well as a proangiogenic manner if released by immature DC in hypoxic tumor sites.

## 6. Myeloid Cell Mediate Resistance to Antiangiogenic Drugs

Recently, Shojaei et al. [78] reported that accumulation of CD11b^+^Gr1^+^ cells in tumors renders their refractory to anti-angiogenic blockage by VEGF antibodies. Different murine tumor cell lines were tested for their responsiveness to anti-VEGF antibody treatment. Refractory tumors were associated with significant increase in the frequency of tumor infiltrating CD11b^+^Gr1^+^ cells compared to sensitive tumors. Moreover, when normally sensitive tumor cells were mixed with these cells that are resistant to anti-VEGF antibodies and transplanted into other mice, the transplanted tumors resist anti-VEGF antibodies. In contrast, CD11b^+^Gr1^+^ cells isolated from sensitive tumors were unable to mediate refractoriness to anti-VEGF treatment, indicating that the tumor directly modulates CD11b^+^Gr1^+^ cells to promote angiogenesis independent of VEGF. Gene array analysis revealed an upregulation of G-CSF and monocyte chemotactic protein 1 (MCP-1) in resistant tumors, both factors known to be involved in the mobilization of bone marrow derived myeloid cells to the peripheral blood. In addition, proinflammatory factors such as macrophage inflammatory protein 2 (MIP-2), IL-1 inducible protein, and IL-1*β* were also upregulated in resistant tumors, while resistant-mediating CD11b^+^Gr1^+^ cells revealed increased expression of proinflammatory cytokine receptors such as IL-1, IL-4, IL-11, and IL-13. Taken together, these findings suggest that inflammation is an important aspect of tumor refractoriness in response to anti-VEGF antibody treatment.

Fischer et al., [79] described the use of neutralizing murine antiplacental growth factor (PlGF) monoclonal antibody [79]. Anti-PlGF antibody inhibited growth and metastasis of various tumors, including those resistant to VEGF-receptor 2 (VEGFR2) inhibitors. In contrast to anti-VEGFR2 treatment, anti-PlGF prevented infiltration of angiogenic macrophages and severe tumor hypoxia and, thus, did not switch on the “angiogenic rescue program” which is considered to be responsible for the resistance to anti-VEGFR2 treatment.

## 7. Mobilization and Recruitment of Myeloid Cells into Tumors

Substantial evidence indicates that myeloid cells and their precursors promote neovascularization in tumors and inflammatory tissues. These cells are actively recruited to the tumor microenvironment from the bloodstream. Immune cell trafficking in vivo is regulated by chemokines and by members of the integrin, immunoglobulin superfamily, and selectin adhesion molecule families [80, 81]. Hypoxia, as well as chemokines and their receptors, stimulates homing of circulating myeloid cells to tissues. When tumors encounter low oxygen tension, they adapt by promoting expression of genes associated with angiogenesis, metastasis, and invasion. This transcriptional response pathway is mediated to a large extent by the dimeric transcription factor complexes of hypoxia-inducible factors (HIFs) [82]. HIF1 activity promotes neovascularization by the induction of variety of proangiogenic factors like VEGF-A, VEGFR1, PDGF-B, bFGF, and angiopoietins that stimulate new blood vessel formation within hypoxic areas. In addition, HIF activity also regulates the expression of several chemoattractant factors, including MCP-1, CSF-1, VEGF-A, TNF*α*, and SDF-1*α*, each of them capable to attract myeloid cells to invade hypoxic tissues [83]. 

MCP-1 (or CCL2) and RANTES (or CCL5) increased the infiltration of TAM into primary tumors, including breast and ovarian carcinomas, melanoma, and glioblastoma [84–87]. Furthermore, MCP-1 and RANTES stimulate the secretion of matrix-degrading enzymes, such as MMP9 and MMP12 by macrophages.

IL-8 also serves as a monocyte chemoattractant. This chemokine is also a proangiogenic factor and an autocrine growth factor for several human tumor cell types [88]. IL-8 stimulates the adhesion of monocytes, which express low levels of the IL-8 receptors CXCR1 and CXCR2, to vascular endothelium under flow conditions. These studies indicate that IL-8 and CXCR-1/2 interactions play roles in monocyte recruitment. Several cytokines and growth factors, including colony stimulating factor-1 (CSF-1), VEGF, and PDGF, have been implicated in the recruitment of monocytes into tumors [89–91]. CSF-1 is produced by various types of human tumors and is a potent chemoattractant for macrophages. Coordinated expression of CSF-1 in macrophages and epidermal growth factor (EGF) in mammary tumor cells resulted in increased myeloid cell invasion into mammary tumors [91].

IL-1*β*, another myeloid cell cytokine, increased infiltration of neutrophils and macrophages in a mouse model of corneal neovascularization. In contrast, deletion of monocytes by genetic approaches or by use of toxins significantly suppressed IL-1*β* induced angiogenesis [92]. 

*β*-defensin may also serve as a recruitment factor for myeloid lineage cells. *β*-defensin is a chemoattractant factor for DC. Conjeo-Garcia and colleagues found that the recruitment of dendritic precursor cells into tumors required the presence of *β*-defensin [74]. Depletion of *β*-defensin or inhibition of its receptor CCR6 using function-blocking antibodies abolished the infiltration of dendritic precursor cells into tumors. These studies indicate that the ligand/receptor pair *β*-defensin/CCR6 is essential for dendritic precursor cell recruitment. 

A key role for SDF-1*α* in progenitor cell recruitment was recently described [93]. Syngeneic tumors transplanted into thrombocytopenic mice (such as Thpo-/- and Mpl-/- mice) exhibited impaired neovascularization and reduced release of the chemokine SDF-1*α*. Further studies demonstrated that hematopoietic cytokines including soluble Kit-ligand and thrombopoietin trigger the release of SDF-1*α* from platelets, which results in the mobilization of unique subset of hemangiogenic progenitor cells (CXCR4+ VEGFR1+) to neoangiogenic niches. 

Du et al., [9] recently reported that HIF-1*α*, the direct effector of hypoxia, induces recruitment of diverse bone marrow derived subpopulations, containing Tie2^+^, VEGFR1^+^, CD11b^+^, and F4/80^+^, as well as EPC and pericyte progenitors to promote neovascularization in glioblastoma. HIF-1*α* contributed to the induction of SDF-1*α* in glioblastoma cells, which in turn promoted tumor progression by recruiting MMP9^+^ vascular modulatory bone marrow cells [9]. 

Recently Bv8, also known as prokineticin-2, was identified as a critical regulator for CD11b^+^Gr1^+^-mediated angiogenesis. Bv8 and the related EG-VEGF were also characterized as mitogens for specific endothelial cell types [94]. Both Bv8 and EG-VEGF bind two highly homologous G-protein-coupled receptors termed PKR-1 and PKR2. Bv8 expression was reported to be upregulated in CD11b^+^Gr1^+^ cells after tumor implantation [8]. Bv8 was shown to mobilize hematopoietic cells such as CD11b^+^Gr1^+^ cells to the blood and also stimulated the production of granulocytic and monocytic colonies in vitro [95]. Notably, anti-Bv8 treatment of mice implanted with human tumors resulted in a significant reduction in tumor growth and tumor angiogenesis. This effect was associated with a reduction in CD11b^+^Gr1^+^ mobilization from the bone marrow. Interestingly G-CSF dramatically upregulates Bv8 expression [8, 96]. Hence, G-CSF produced by the tumor cells or tumor associated fibroblast may result in an upregulation of Bv8 in the BM, which, in turn, results in the induction of differentiation of myeloid progenitors and their mobilization to the peripheral blood.

The immune suppressive molecule TGF-*β* has also been implicated in myeloid cell functions. Experiments with a transplanted and spontaneous mammary carcinoma demonstrated increased levels of TGF-*β* in the tumor microenvironment if the tumor cells were deficient for the type II TGF-*β* receptor (*Tgfbr2* KO). These authors demonstrated that a deficiency in the receptor resulted in an increase in CXCL5 (ENA-78) and SDF-1*α* in the tumor microenvironment. Further analysis targeting the CXCL5 receptor CXCR2 with antagonist decreased the recruitment of CD11b^+^Gr1^+^ cells to orthotopic transplanted *Tgfbr2* KO breast adenocarcinomas [97]. 

These studies demonstrate that a variety of inflammatory stimuli can recruit diverse subsets of myeloid cells to invade tumor tissue. 

## 8. Conclusions

**Figure 2:**
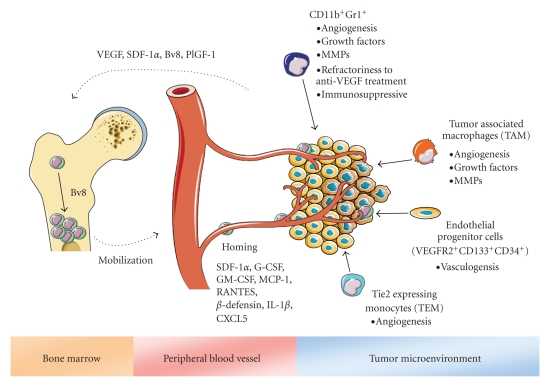
Recruitment of diverse bone marrow-derived cell populations to the tumor microenvironment and their effects on tumor progression. Tumor and stromal cells mobilize various subpopulations of tumor promoting bone marrow-derived cells to the peripheral blood through secretion of cytokines and chemokines. Diverse chemoattractant factors promote the recruitment and infiltration of these cells to the tumor microenvironment where they suppress the antitumor immunity or promote tumor angiogenesis and vasculogenesis or raise refractoriness against anti-VEGF therapy.

Links between chronic inflammation and cancer have been recognized for several decades. Studies to understand the recruitment of proangiogenic myeloid cells populations and immunsuppressive MDSC and their contributions to angiogenesis are ongoing. All these studies suggest that several myeloid subpopulations may play roles during neovascularization of tumors, mediating refractoriness to anti-angiogenic therapies, or the escape from immune surveillance (Figure 2). Much progress is needed with regards to the characterization of markers to identify cells subsets that have specific regulatory roles. This might further help to understand why so many different characterized cell types appear to have overlapping functions. 

Myeloid cells represent novel targets for therapeutic strategies. The mobilization and recruitment of myeloid cells by the tumor defines myeloid cells as a potential delivery system to target the tumor microenvironment. One such approach was recently shown using TEM. Mice transplanted with TEM expressing interferon *α* (IFN-*α*), a potent cytokine with angiostatic and anti-proliferative activity [98] under the *Tie2* promotor, inhibited tumor progression in several tumor models [99]. Targeting cytokines and cytotoxic proteins to tumors by means of gene-modified myeloid cells thus represents a promising strategy to treat cancer [100]. 

In contrast, the tumor promoting properties of myeloid cells define these cells as putative targets for anticancer therapies. Anti-angiogenic agents were already described to be the most efficacious when combined with cytotoxic agents and/or therapies targeted towards ablating myeloid cells [5, 101]. Furthermore, suppression of myeloid cell recruitment to the tumor microenvironment offers a new strategy to inhibit tumor neovascularization, while stimulation of homing may promote tissue recovery from ischemia.

## References

[1] Carmeliet P (2005). Angiogenesis in life, disease and medicine. *Nature*.

[2] Coussens LM, Werb Z (2002). Inflammation and cancer. *Nature*.

[3] Folkman J (1971). Tumor angiogenesis: therapeutic implications. *The New England Journal of Medicine*.

[4] Hanahan D, Folkman J (1996). Patterns and emerging mechanisms of the angiogenic switch during tumorigenesis. *Cell*.

[5] Kerbel RS (2008). Tumor angiogenesis. *The New England Journal of Medicine*.

[6] Folkman J, Merler E, Abernathy C, Williams G (1971). Isolation of a tumor factor responsible or angiogenesis. *Journal of Experimental Medicine*.

[7] Carmeliet P, Jain RK (2000). Angiogenesis in cancer and other diseases. *Nature*.

[8] Shojaei F, Wu X, Zhong C (2007). Bv8 regulates myeloid-cell-dependent tumour angiogenesis. *Nature*.

[9] Du R, Lu KV, Petritsch C (2008). HIF1*α* induces the recruitment of bone marrow-derived vascular modulatory cells to regulate tumor angiogenesis and invasion. *Cancer Cell*.

[10] DeNardo DG, Barreto JB, Andreu P (2009). CD4^+^ T cells regulate pulmonary metastasis of mammary carcinomas by enhancing protumor properties of macrophages. *Cancer Cell*.

[11] Fridlender ZG, Sun J, Kim S (2009). Polarization of tumor-associated neutrophil phenotype by TGF-*β*: “N1” versus “N2” TAN. *Cancer Cell*.

[12] Schmid MC, Varner JA (2007). Myeloid cell trafficking and tumor angiogenesis. *Cancer Letters*.

[13] Asahara T, Murohara T, Sullivan A (1997). Isolation of putative progenitor endothelial cells for angiogenesis. *Science*.

[14] Peichev M, Naiyer AJ, Pereira D (2000). Expression of VEGFR-2 and AC133 by circulating human CD34^+^ cells identifies a population of functional endothelial precursors. *Blood*.

[15] Gill M, Dias S, Hattori K (2001). Vascular trauma induces rapid but transient mobilization of VEGFR2^+^AC133^+^ endothelial precursor cells. *Circulation Research*.

[16] Gao D, Nolan D, McDonnell K (2009). Bone marrow-derived endothelial progenitor cells contribute to the angiogenic switch in tumor growth and metastatic progression. *Biochimica et Biophysica Acta*.

[17] Gao D, Nolan DJ, Mellick AS, Bambino K, McDonnell K, Mittal V (2008). Endothelial progenitor cells control the angiogenic switch in mouse lung metastasis. *Science*.

[18] Garcia-Barros M, Paris F, Cordon-Cardo C (2003). Tumor response to radiotherapy regulated by endothelial cell apoptosis. *Science*.

[19] Spring H, Schuler T, Arnold B, Hammerling GJ, Ganss R (2005). Chemokines direct endothelial progenitors into tumor neovessels. *Proceedings of the National Academy of Sciences of the United States of America*.

[20] Peters BA, Diaz LA, Polyak K (2005). Contribution of bone marrow-derived endothelial cells to human tumor vasculature. *Nature Medicine*.

[21] Mantovani A, Sozzani S, Locati M, Allavena P, Sica A (2002). Macrophage polarization: tumor-associated macrophages as a paradigm for polarized M2 mononuclear phagocytes. *Trends in Immunology*.

[22] Mantovani A, Schioppa T, Porta C, Allavena P, Sica A (2006). Role of tumor-associated macrophages in tumor progression and invasion. *Cancer and Metastasis Reviews*.

[23] Martinez FO, Helming L, Gordon S (2009). Alternative activation of macrophages: an immunologic functional perspective. *Annual Review of Immunology*.

[24] Martinez FO, Sica A, Mantovani A, Locati M (2008). Macrophage activation and polarization. *Frontiers in Bioscience*.

[25] Biswas SK, Sica A, Lewis CE (2008). Plasticity of macrophage function during tumor progression: regulation by distinct molecular mechanisms. *Journal of Immunology*.

[26] Lewis JS, Landers RJ, Underwood JCE, Harris AL, Lewis CE (2000). Expression of vascular endothelial growth factor by macrophages is up-regulated in poorly vascularized areas of breast carcinomas. *Journal of Pathology*.

[27] Sunderkotter C, Steinbrink K, Goebeler M, Bhardwaj R, Sorg C (1994). Macrophages and angiogenesis. *Journal of Leukocyte Biology*.

[28] Giraudo E, Inoue M, Hanahan D (2004). An amino-bisphosphonate targets MMP-9—expressing macrophages and angiogenesis to impair cervical carcinogenesis. *Journal of Clinical Investigation*.

[29] Hildenbrand R, Dilger I, Horlin A, Stutte HJ (1995). Urokinase and macrophages in tumour angiogenesis. *British Journal of Cancer*.

[30] Balkwill F, Mantovani A (2001). Inflammation and cancer: back to Virchow?. *The Lancet*.

[31] Huang S, Van Arsdall M, Tedjarati S (2002). Contributions of stromal metalloproteinase-9 to angiogenesis and growth of human ovarian carcinoma in mice. *Journal of the National Cancer Institute*.

[32] Esposito I, Menicagli M, Funel N (2004). Inflammatory cells contribute to the generation of an angiogenic phenotype in pancreatic ductal adenocarcinoma. *Journal of Clinical Pathology*.

[33] Polverini PJ, Leibovich SJ (1987). Effect of macrophage depletion on growth and neovascularization of hamster buccal pouch carcinomas. *Journal of Oral Pathology*.

[34] Leek RD, Harris AL (2002). Tumor-associated macrophages in breast cancer. *Journal of Mammary Gland Biology and Neoplasia*.

[35] Nishie A, Ono M, Shono T (1999). Macrophage infiltration and heme oxygenase-1 expression correlate with angiogenesis in human gliomas. *Clinical Cancer Research*.

[36] Gabrilovich DI, Nagaraj S (2009). Myeloid-derived suppressor cells as regulators of the immune system. *Nature Reviews Immunology*.

[37] Yang R, Cai Z, Zhang Y, Yutzy WH, Roby KF, Roden RBS (2006). CD80 in immune suppression by mouse ovarian carcinoma-associated Gr-1^+^CD11b^+^ myeloid cells. *Cancer Research*.

[38] Gallina G, Dolcetti L, Serafini P (2006). Tumors induce a subset of inflammatory monocytes with immunosuppressive activity on CD8^+^ T cells. *Journal of Clinical Investigation*.

[39] Youn J-I, Nagaraj S, Collazo M, Gabrilovich DI (2008). Subsets of myeloid-derived suppressor cells in tumor-bearing mice. *Journal of Immunology*.

[40] Sawanobori Y, Ueha S, Kurachi M (2008). Chemokine-mediated rapid turnover of myeloid-derived suppressor cells in tumor-bearing mice. *Blood*.

[41] Ochoa AC, Zea AH, Hernandez C, Rodriguez PC (2007). Arginase, prostaglandins, and myeloid-derived suppressor cells in renal cell carcinoma. *Clinical Cancer Research*.

[42] Almand B, Clark JI, Nikitina E (2001). Increased production of immature myeloid cells in cancer patients: a mechanism of immunosuppression in cancer. *Journal of Immunology*.

[43] Schmielau J, Finn OJ (2001). Activated granulocytes and granulocyte-derived hydrogen peroxide are the underlying mechanism of suppression of T-cell function in advanced cancer patients. *Cancer Research*.

[44] Kusmartsev S, Gabrilovich DI (2006). Role of immature myeloid cells in mechanisms of immune evasion in cancer. *Cancer Immunology, Immunotherapy*.

[45] Rodriguez PC, Ernstoff MS, Hernandez C (2009). Arginase I-producing myeloid-derived suppressor cells in renal cell carcinoma are a subpopulation of activated granulocytes. *Cancer Research*.

[46] Nagaraj S, Gupta K, Pisarev V (2007). Altered recognition of antigen is a mechanism of CD8^+^ T cell tolerance in cancer. *Nature Medicine*.

[47] Sauer H, Wartenberg M, Hescheler J (2001). Reactive oxygen species as intracellular messengers during cell growth and differentiation. *Cellular Physiology and Biochemistry*.

[48] Taketo MM (1998). Cyclooxygenase-2 inhibitors in tumorigenesis (part I). *Journal of the National Cancer Institute*.

[49] Bertagnolli MM (2007). Chemoprevention of colorectal cancer with cyclooxygenase-2 inhibitors: two steps forward, one step back. *The Lancet Oncology*.

[50] Ostrand-Rosenberg S, Sinha P (2009). Myeloid-derived suppressor cells: linking inflammation and cancer. *Journal of Immunology*.

[51] Fichtner-Feigl S, Terabe M, Kitani A (2008). Restoration of tumor immunosurveillance via targeting of interleukin-13 receptor-*α*2. *Cancer Research*.

[52] Terabe M, Matsui S, Park J-M (2003). Transforming growth factor-beta production and myeloid cells are an effector mechanism through which CD1d-restricted T cells block cytotoxic T lymphocyte-mediated tumor immunosurveillance: abrogation prevents tumor recurrence. *Journal of Experimental Medicine*.

[53] De Palma M, Venneri MA, Galli R (2005). Tie2 identifies a hematopoietic lineage of proangiogenic monocytes required for tumor vessel formation and a mesenchymal population of pericyte progenitors. *Cancer Cell*.

[54] De Palma M, Venneri MA, Naldini L (2003). In vivo targeting of tumor endothelial cells by systemic delivery of lentiviral vectors. *Human Gene Therapy*.

[55] De Palma M, Naldini L (2009). Tie2-expressing monocytes (TEMs): novel targets and vehicles of anticancer therapy?. *Biochimica et Biophysica Acta*.

[56] Eck M, Schmausser B, Scheller K, Brandlein S, Muller-Hermelink HK (2003). Pleiotropic effects of CXC chemokines in gastric carcinoma: differences in CXCL8 and CXCL1 expression between diffuse and intestinal types of gastric carcinoma. *Clinical and Experimental Immunology*.

[57] Bellocq A, Antoine M, Flahault A (1998). Neutrophil alveolitis in bronchioloalveolar carcinoma: induction by tumor-derived interleukin-8 and relation to clinical outcome. *American Journal of Pathology*.

[58] Bergers G, Brekken R, McMahon G (2000). Matrix metalloproteinase-9 triggers the angiogenic switch during carcinogenesis. *Nature Cell Biology*.

[59] McCourt M, Wang JH, Sookhai S, Redmond HP (1999). Proinflammatory mediators stimulate neutrophil-directed angiogenesis. *Archives of Surgery*.

[60] Rothenberg ME, Hogan SP (2006). The eosinophil. *Annual Review of Immunology*.

[61] Dorta RG, Landman G, Kowalski LP, Lauris JRP, Latorre MR, Oliveira DT (2002). Tumour-associated tissue eosinophilia as a prognostic factor in oral squamous cell carcinomas. *Histopathology*.

[62] Looi L-M (1987). Tumor-associated tissue eosinophilia in nasopharyngeal carcinoma. A pathologic study of 422 primary and 138 metastatic tumors. *Cancer*.

[63] Teruya-Feldstein J, Jaffe ES, Burd PR, Kingma DW, Setsuda JE, Tosato G (1999). Differential chemokine expression in tissues involved by Hodgkin’s disease: direct correlation of eotaxin expression and tissue eosinophilia. *Blood*.

[64] Nielsen HJ, Hansen U, Christensen IJ, Reimert CM, Brunner N, Moesgaard F (1999). Independent prognostic value of eosinophil and mast cell infiltration in colorectal cancer tissue. *Journal of Pathology*.

[65] Lorena SCM, Oliveira DT, Dorta RG, Landman G, Kowalski LP (2003). Eotaxin expression in oral squamous cell carcinomas with and without tumour associated tissue eosinophilia. *Oral Diseases*.

[66] Mattes J, Hulett M, Xie W (2003). Immunotherapy of cytotoxic T cell-resistant tumors by T helper 2 cells: an eotaxin and STAT6-dependent process. *Journal of Experimental Medicine*.

[67] Horiuchi T, Weller PF (1997). Expression of vascular endothelial growth factor by human eosinophils: upregulation by granulocyte macrophage colony-stimulating factor and interleukin-5. *American Journal of Respiratory Cell and Molecular Biology*.

[68] Cormier SA, Taranova AG, Bedient C (2006). Pivotal advance: eosinophil infiltration of solid tumors is an early and persistent inflammatory host response. *Journal of Leukocyte Biology*.

[69] Ribatti D, Crivellato E (2009). The controversial role of mast cells in tumor growth. *International Review of Cell and Molecular Biology*.

[70] Crivellato E, Nico B, Ribatti D (2008). Mast cells and tumour angiogenesis: new insight from experimental carcinogenesis. *Cancer Letters*.

[71] Colonna M, Trinchieri G, Liu Y-J (2004). Plasmacytoid dendritic cells in immunity. *Nature Immunology*.

[72] Gottfried E, Kreutz M, Mackensen A (2008). Tumor-induced modulation of dendritic cell function. *Cytokine and Growth Factor Reviews*.

[73] Fricke I, Gabrilovich DI (2006). Dendritic cells and tumor microenvironment: a dangerous liaison. *Immunological Investigations*.

[74] Conejo-Garcia JR, Benencia F, Courreges M-C (2004). Tumor-infiltrating dendritic cell precursors recruited by a *β*-defensin contribute to vasculogenesis under the influence of Vegf-A. *Nature Medicine*.

[75] Coukos G, Conejo-Garcia JR, Buckanovich R, Benencia F (2007). Vascular leukocytes: a population with angiogenic and immunossuppressive properties highly represented in ovarian cancer. *Advances in Experimental Medicine and Biology*.

[76] Fainaru O, Almog N, Yung CW (2010). Tumor growth and angiogenesis are dependent on the presence of immature dendritic cells. *The FASEB Journal*.

[77] Ricciardi A, Elia AR, Cappello P (2008). Transcriptome of hypoxic immature dendritic cells: modulation of chemokine/receptor expression. *Molecular Cancer Research*.

[78] Shojaei F, Wu X, Malik AK (2007). Tumor refractoriness to anti-VEGF treatment is mediated by CD11b^+^Gr1^+^ myeloid cells. *Nature Biotechnology*.

[79] Fischer C, Jonckx B, Mazzone M (2007). Anti-PlGF inhibits growth of VEGF(R)-inhibitor-resistant tumors without affecting healthy vessels. *Cell*.

[80] Weber C, Koenen RR (2006). Fine-tuning leukocyte responses: towards a chemokine ‘interactome’. *Trends in Immunology*.

[81] Luster AD, Alon R, von Andrian UH (2005). Immune cell migration in inflammation: present and future therapeutic targets. *Nature Immunology*.

[82] Giaccia AJ, Simon MC, Johnson R (2004). The biology of hypoxia: the role of oxygen sensing in development, normal function, and disease. *Genes and Development*.

[83] Liao D, Johnson RS (2007). Hypoxia: a key regulator of angiogenesis in cancer. *Cancer and Metastasis Reviews*.

[84] Ueno T, Toi M, Saji H (2000). Significance of macrophage chemoattractant protein-1 in macrophage recruitment, angiogenesis, and survival in human breast cancer. *Clinical Cancer Research*.

[85] Niwa Y, Akamatsu H, Niwa H, Sumi H, Ozaki Y, Abe A (2001). Correlation of tissue and plasma RANTES levels with disease course in patients with breast or cervical cancer. *Clinical Cancer Research*.

[86] Lin EY, Nguyen AV, Russell RG, Pollard JW (2001). Colony-stimulating factor 1 promotes progression of mammary tumors to malignancy. *Journal of Experimental Medicine*.

[87] Murdoch C, Giannoudis A, Lewis CE (2004). Mechanisms regulating the recruitment of macrophages into hypoxic areas of tumors and other ischemic tissues. *Blood*.

[88] Zhu YM, Webster SJ, Flower D, Woll PJ (2004). Interleukin-8/CXCL8 is a growth factor for human lung cancer cells. *British Journal of Cancer*.

[89] Uutela M, Wirzenius M, Paavonen K (2004). PDGF-D induces macrophage recruitment, increased interstitial pressure, and blood vessel maturation during angiogenesis. *Blood*.

[90] Barleon B, Sozzani S, Zhou D, Weich HA, Mantovani A, Marme D (1996). Migration of human monocytes in response to vascular endothelial growth factor (VEGF) is mediated via the VEGF receptor flt-1. *Blood*.

[91] Goswami S, Sahai E, Wyckoff JB (2005). Macrophages promote the invasion of breast carcinoma cells via a colony-stimulating factor-1/epidermal growth factor paracrine loop. *Cancer Research*.

[92] Nakao S, Kuwano T, Tsutsumi-Miyahara C (2005). Infiltration of COX-2-expressing macrophages is a prerequisite for IL-1*β*-induced neovascularization and tumor growth. *Journal of Clinical Investigation*.

[93] Jin DK, Shido K, Kopp H-G (2006). Cytokine-mediated deployment of SDF-1 induces revascularization through recruitment of CXCR4^+^ hemangiocytes. *Nature Medicine*.

[94] LeCouter J, Kowalski J, Foster J (2001). Identification of an angiogenic mitogen selective for endocrine gland endothelium. *Nature*.

[95] LeCouter J, Zlot C, Tejada M, Peale F, Ferrara N (2004). Bv8 and endocrine gland-derived vascular endothelial growth factor stimulate hematopoiesis and hematopoietic cell mobilization. *Proceedings of the National Academy of Sciences of the United States of America*.

[96] Shojaei F, Wu X, Qu X (2009). G-CSF-initiated myeloid cell mobilization and angiogenesis mediate tumor refractoriness to anti-VEGF therapy in mouse models. *Proceedings of the National Academy of Sciences of the United States of America*.

[97] Yang L, Huang J, Ren X (2008). Abrogation of TGF*β* signaling in mammary carcinomas recruits Gr-1^+^CD11b^+^ myeloid cells that promote metastasis. *Cancer Cell*.

[98] Stark GR, Kerr IM, Williams BRG, Silverman RH, Schreiber RD (1998). How cells respond to interferons. *Annual Review of Biochemistry*.

[99] De Palma M, Mazzieri R, Politi LS (2008). Tumor-targeted interferon-*α* delivery by Tie2-expressing monocytes inhibits tumor growth and metastasis. *Cancer Cell*.

[100] Aboody KS, Najbauer J, Danks MK (2008). Stem and progenitor cell-mediated tumor selective gene therapy. *Gene Therapy*.

[101] Ferrara N, Kerbel RS (2005). Angiogenesis as a therapeutic target. *Nature*.

